# Dealing with the Effects of Sensor Displacement in Wearable Activity Recognition

**DOI:** 10.3390/s140609995

**Published:** 2014-06-06

**Authors:** Oresti Banos, Mate Attila Toth, Miguel Damas, Hector Pomares, Ignacio Rojas

**Affiliations:** 1 Department of Computer Architecture and Computer Technology, Research Center for Information and Communications Technologies-University of Granada (CITIC-UGR), C/Calle Periodista Rafael Gomez Montero 2, Granada E18071, Spain; E-Mails: mdamas@ugr.es (M.D.); hector@ugr.es (H.P.); irojas@ugr.es (I.R.); 2 Language and Speech Laboratory, University of the Basque Country, Paseo de la Universidad 5, Vitoria E01006, Spain; E-Mail: a.m.toth@laslab.org

**Keywords:** activity recognition, sensor displacement, wearable sensors, inertial sensing, sensor fusion, human behavior inference, real-world

## Abstract

Most wearable activity recognition systems assume a predefined sensor deployment that remains unchanged during runtime. However, this assumption does not reflect real-life conditions. During the normal use of such systems, users may place the sensors in a position different from the predefined sensor placement. Also, sensors may move from their original location to a different one, due to a loose attachment. Activity recognition systems trained on activity patterns characteristic of a given sensor deployment may likely fail due to sensor displacements. In this work, we innovatively explore the effects of sensor displacement induced by both the intentional misplacement of sensors and self-placement by the user. The effects of sensor displacement are analyzed for standard activity recognition techniques, as well as for an alternate robust sensor fusion method proposed in a previous work. While classical recognition models show little tolerance to sensor displacement, the proposed method is proven to have notable capabilities to assimilate the changes introduced in the sensor position due to self-placement and provides considerable improvements for large misplacements.

## 1. Introduction

The technologies of daily living are devised to facilitate users' everyday activities and to safeguard their wellbeing. In the past couple of decades, thousands of products have been released to make our life easier, safer and more comfortable, allowing us to concentrate on more important tasks. Wearable technology emerges in this context to assist users in their daily tasks in a transparent manner. To that end, sensors and systems are integrated into articles of everyday use, principally embedded in accessories. However, transparency is not only achieved through concealing technology in a physical manner, but it is also achieved when no specific attention is required from the wearer during use. In fact, on-body systems cease to be transparent when users start to need to pay attention to the way they are worn, for example, when a bracelet must be placed on a specific limb or a watch positioned in a determined orientation.

Current activity recognition systems require sensors to be located at predetermined positions during use to successfully discriminate between activities [1–4]. Pattern models are derived in a training step before the system deployment, where the sensor positions are considered to be constant. In particular, for on-body sensors, a constant position on the body cannot be maintained in real-life scenarios [5]. Sensor deployments are subject to variations introduced by the normal use of the accessories into which they are embedded. These variations correspond to sensor position changes or displacements on the user's body. Displacements can remain static during the execution of many activity instances, e.g., when sensors are initially misplaced and remain that way throughout the day [6]. Sensors may also change position dynamically due to loose attachments, e.g., when integrated into baggy clothes [7]. The effect of placement-related changes on sensor measurements is profound. Sensor data distributions can drastically drift from the activity patterns observed with the sensors in the default positions. As a consequence, previously trained pattern models may fail to identify target activities in the observed sensor data.

In this work we evaluate the effects of sensor displacement on activity recognition using standard techniques for different settings. The results are compared to a sensor fusion method proposed in a previous work, designed to cope with the effects of sensor displacement. The rest of the paper is structured as follows. Section 2 presents prior works dealing with sensor displacement in on-body activity recognition. In Section 3, the concept of sensor displacement is presented, defined as a combination of rotations and translations. This section also illustrates the principal effects of sensor displacement on inertial sensing modalities. Section 4 describes an innovative dataset, which is used to benchmark activity recognition models under the presence of sensor displacement measured in realistic settings. In Section 5, some of the most predominant activity recognition models are evaluated for several sensor displacement scenarios, as well as an alternative method proposed in a previous work to cope with similar sensor anomalies. The obtained results are subsequently discussed in Section 6, while our final conclusions are summarized in Section 7.

## 2. State-of-the-Art

Diverse approaches have been proposed in the literature to increase the robustness of on-body inertial sensor-based activity recognition systems against sensor displacement. One main approach to deal with this issue consists of identifying displacement-invariant features for recognition. Kunze *et al.* [8] studied how acceleration and gyroscope signals are affected by sensor displacement. They distinguished between gravitational, translational and rotational components in the acceleration signal and showed that the acceleration component due to rotation is especially sensitive to sensor displacement. Based on this observation, they proposed a heuristic method, which achieved higher recognition rates for sensor displacement within a particular body part. Another approach that uses displacement-invariant features was proposed by Foerster *et al.* [9]. By extracting signals from several locations within a body segment and applying a genetic algorithm for feature selection, they identified features invariant to sensor displacement. They validated their approach using a human-computer interaction (HCI) gesture dataset and a fitness dataset and achieved improved recognition results with respect to standard features. These heuristic methods are coupled to the assumption that the user performs the required specific activities at some point, which nevertheless might not be always guaranteed.

Another main direction to increase recognition robustness against sensor displacement is to adapt the classification algorithm to the resulting shifts in the signal and feature distribution. Gao *et al.* [10] utilize an estimate of the constant gravity vector to transform the accelerometer signals from the device coordinate system, which is sensitive to the orientation, to the body reference coordinate system, which is seen to be steady. This estimation is only possible when the user remains static. The approach is evaluated for four categories of activities (lying down, sitting, standing and walking) demonstrating an increase of the overall accuracy when using the body reference frame with respect to the case in which the sensor's original frame is considered. In [11], a calibration matrix calculated using Gram–Schmidt orthonormalization is applied to eliminate sensor orientation errors, while a low-pass filter is employed to remove the main effect of sensor misplacement. A support vector machine (SVM) classifier is implemented to recognize human activities by using the calibrated acceleration data. A similar calibration process is proposed in [12], which consists of the rectification of the signals registered through displaced sensors from the analysis of repetitive motion signatures. Very promising results were obtained in combination with dynamic time warping (DTW), albeit only for the activity, walking. In a previous work, Kale *et al.* [13] showed the tolerance of recognition systems using DTW to small sensor misplacements and rotations. In [14], the robust motion direction of the user is obtained for sensors prone to rotation by using models based on principal component analysis (PCA). At the feature level, [15,16] proposed an unsupervised adaptation method based on the expectation-maximization (EM) algorithm. They assumed that the anomalies introduced by sensor displacements can be characterized as a covariate shift [17]. They estimated this shift using an online version of the EM algorithm and transformed the sensor readings back in the feature space before classification. They tested their method on HCI, fitness and daily living scenarios. While the previous methods applied a transformation in the signal or feature space, in [18], the authors proposed an online self-calibration method to dynamically adapt the classifier model. The method consists of a calibration phase and a recognition phase. The calibration phase is triggered by the user when they observe that the recognition accuracy drops. In this phase, the cluster centers of a nearest centroid classifier (NCC) are adjusted at a predefined learning rate using the incoming instances after classification. The process is stopped when the gradient of the distance between the adapted class center and the original class center drops below a certain threshold. This method is intrusive and depends on the capacity of the user to identify erroneous recognition.

Multi-sensor fusion has been also utilized in the past for counteracting the effects of sensor orientation changes. In [19], the authors showed a significant tolerance increase by using a large set of sensors in combination with majority voting or naive Bayes decision fusion models. A more sophisticated scheme is presented in [20], which attempts to detect anomalies and potentially affected sensors in order to remove them from the sensor ecology. This approach is further improved in [21] by bringing the system to a new stable state. This is accomplished through a self-training process, which uses the fusion output as labels to retrain the sensor systems identified as anomalous. A novel sensor fusion model is also proposed in [22] for dealing with sensor displacement. This model proved to satisfactorily cope with slight to moderate variations for any number of displaced sensors and to be also good enough when a minority of the sensors are largely modified with respect to their original positions. For all of these works, the methods are validated on synthetically introduced rotations; however, an evaluation of the effects of translational sensor displacement is usually lacking. More importantly, the analysis of sensor displacement in a realistic scenario is practically unprecedented.

## 3. Sensor Displacement

Sensor displacement can be defined as a change in sensor position with respect to the initial or intended position during use. In on-body sensing, sensor displacement is observed when a sensor mounted on a given body part is moved to another body location, which may be close to (small to moderate displacement) or distant (extreme mispositioning) from the initial placement. Displacement-related sensor anomalies are seen to be highly relevant for inertial sensors and, thus, in this work, profoundly analyzed.

A common approximation used in biomechanics or human body motion modeling consists of rigid segments (mostly representing body limbs), which are connected through joints that allow rotation around one (e.g., elbow) or more (e.g., wrist) axes. Although the human body cannot be literally seen as a rigid body (soft tissue, skin motion, muscle activity), this model fairly approximates most motion interactions and provides a tractable approach for analysis and representation. According to the physics of the rigid body, sensor displacement may be seen as the combination of two transformations: rotations and translations. Rotations refer to the circular movements that the sensor experiences around its rotation axes or upon itself. Translations correspond to the movement of every point of the sensor the same distance in a given direction. While sensor displacement between different limbs are less common in real-life applications, shifts and rotations on the same limb occur frequently.

Sensor displacement affects each inertial sensing modality (acceleration, rate of turn, magnetic field) to a different extent. Thus, for example, acceleration is especially sensitive to rotations. Rotations introduce a change in the sensor local frame of reference with respect to its original spatial distribution. This causes a shift in the direction of the gravitational component with respect to the sensor reference frame. The effect of translations is normally more dependent on the initial and end position, as well as the magnitude of the acceleration experienced by the sensor. Thus, for example, a sensor that is displaced from the upper arm to the lower arm will generally measure higher acceleration. On the contrary, during inactivity or while in an idle state, this change may not be noticeable. More robust to displacement anomalies are gyroscopes, which are minimally affected by rotations along their rotation axis and translations along the same body limb. Gyroscopes do not measure angles directly, but angular velocity, which is integrated to obtain angular positions. In the case of motion, the gyroscope remains unaffected by displacements along a given body limb, since, during a translation or rotation, all points of the rigid body are rotated or moved to a similar extent (same linear and angular velocity/acceleration for all points of the same body limb). However, gyroscopes provide no relevant information when the user remains still, thus proving of little utility to assess sedentary or passive activities. The compass (magnetic field sensor) measurements are also affected by rotations and, to a lower extent, by translations when assuming no gimbal lock degeneration. This phenomena corresponds to the loss of one degree of freedom within a gimbal system-pivoted support that allows the rotation of an object about a single axis-in a three-dimensional space, which occurs when two out of three gimbals line up in a parallel configuration, “locking” the system into a rotation in a degenerated two-dimensional space.

**Figure 1.**
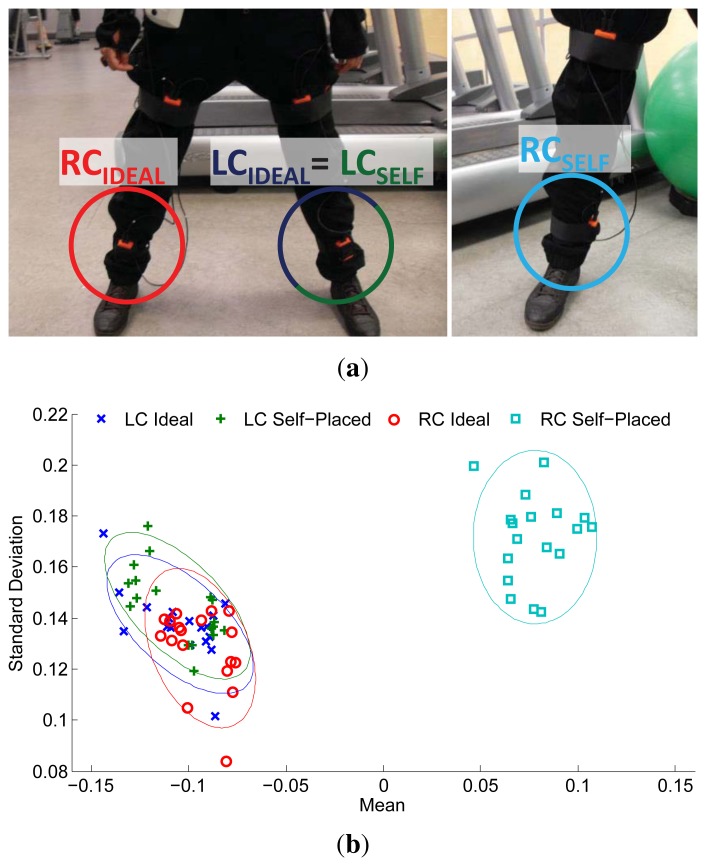
Example of sensor displacement introduced during the user self-placement of a sensor (**a**), and its effect at the feature level (**b**). In this particular example, the displacement with respect to the predefined deployment in the self-placement case applies to the right calf (RC), while the placement remains approximately similar for the sensor attached to the left calf (LC). In (**b**), the mean and standard deviation computed from the sensor acceleration signals is represented for various instances of a given activity. Confidence ellipses are plotted to represent the feature distribution for each case.

Sensor displacement leads to a new sensor position, which results in a change in the signal space. The impact of the displacement on the sensor signal may vary depending on several factors, such as the magnitude of the displacement or the body part considered. Likewise, these displacement effects are also subject to the particular activities, gestures or movements the user performs. For example, a higher acceleration may be measured during running exercises when a sensor is displaced from the upper arm to the wrist; however, for this very case, smaller accelerations could be registered when the user performs strength-training exercises, such as push-ups. In either case, the sensor readings in the new signal space likely differ with respect to those expected from a default or predefined sensor placement. These changes propagate through the different stages of the activity recognition process, thus affecting the inference process. An example of such effects is depicted in Figure 1. Here, a sensor displacement is unintentionally introduced by the user when self-attaching the devices (Figure 1a). This displacement translates into a significant drift at the feature level (Figure 1b). This shift in the feature space complicates the posterior reasoning process. Therefore, a model trained under the assumption of a predefined placement of the sensors (and, accordingly, a bounded feature space) may not correctly operate, due to the variations introduced by the new feature distribution.

## 4. Dataset for Realistic Sensor Displacement

Although there exist multiple datasets to benchmark activity recognition systems [2,23–27], there is no single dataset that widely investigates the effects of sensor displacement. To the best of our knowledge, only a few works approached the problem through “realistic” data; however, these studies only considered a specific body limb and were conducted in lab conditions [18]. These few datasets are further constrained in terms of he number of subjects, activities and even the type of displacement considered, since they focus exclusively on translation. Moreover, they usually lack a realistic user self-placement mode of introducing sensor displacement and solely rely on displacements introduced or “induced” by the expert. In addition, most of these datasets are proprietary and not freely available for use. Thus, a dataset that supports real-world sensor displacement is missing. Here, *REALDISP*, an open-access dataset that includes multiple sensor setups, namely ideal-placement, self-placement and induced-displacement, is presented. The dataset was first introduced in [28] and here described for the interest of this work. The dataset can be downloaded from [29].

**Figure 2.**
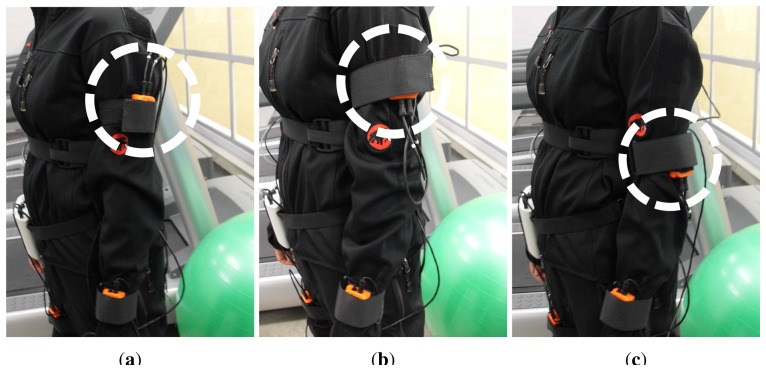
Example of possible sensor placements according to the (**a**) ideal, (**b**) self-placement and (**c**) induced-displacement deployments. In (**b**), the sensor is arbitrarily rotated 180° (approximately) by the user with respect to the ideal positioning (**a**). In (**c**), the expert explicitly displaces the sensor from the middle upper arm to the elbow.

As mentioned previously, a predefined sensor deployment is habitually considered for activity recognition tasks (e.g., Figure 2a). The recognition system is usually trained on this ideal setup. However, users may introduce sensor displacement during self-placement or sensors can drift from their intended position during user activities (e.g., Figure 2b). Larger displacements may be obtained through an intentional mispositioning of the sensors (e.g., Figure 2c), which may be used to investigate the effects of a worst case scenario. To study all these cases, the following three scenarios regarding sensor deployment in real-world settings are defined:

- Ideal-placement or the default scenario. The sensors are positioned by the instructor on predefined locations within each body part. The data stemming from this scenario could be considered as the “training set” for supervised activity recognition systems.
- Self-placement. The user is asked to position a subset of the sensors themselves on the body parts specified by the instructor, but without providing any hint on how the sensors must be exactly placed. This scenario is devised to investigate some of the variability that may occur in the day-to-day usage of an activity recognition system, involving wearable or self-attached sensors. Normally, the self-placement will lead to on-body sensor setups that differ from the ideal-placement. Nevertheless, this difference may be minimal if the subject places the sensor close to the ideal position.
- Induced-displacement. An intentional mispositioning of sensors using rotations and translations with respect to the ideal placement is introduced by the instructor. One of the key interests of including this last scenario is to investigate how the performance of a certain method degrades as the system drifts far from the ideal setup.

The dataset consists of a set of up to 33 typical warm up, fitness and cool down exercises (see Table 1). In particular, the dataset includes activities involving translation (L1–L3), jumps (L4–L8) or general fitness exercises (L31–33), as well as body part-specific activities focused on the trunk (L9–L18), upper extremities (L19–L25) and lower extremities (L26–L30). Diverse reasons support considering this particular activity set. The activities were selected so that different combinations of body parts are involved in each exercise. Some activities imply the motion of the whole body (e.g., walking or jumping), while others focus on training individual parts (e.g., legs for cycling). Since the activities were very easy to perform, participants had no difficulty in doing the exercises. This simplifies the recording process, helps collect large amounts of data and allows users to perform the exercises naturally. The exercise type also influences the impact of the displacement on the sensor signals. Rotation-related anomalies will be constantly present, even when the sensor remains still, due to the orientation drift measured with respect to the gravitational component for accelerometers. On the other hand, translation-related anomalies might only be observable for a given activity when the body part on which the sensor is attached is in motion during the exercise.

**Figure 3.**
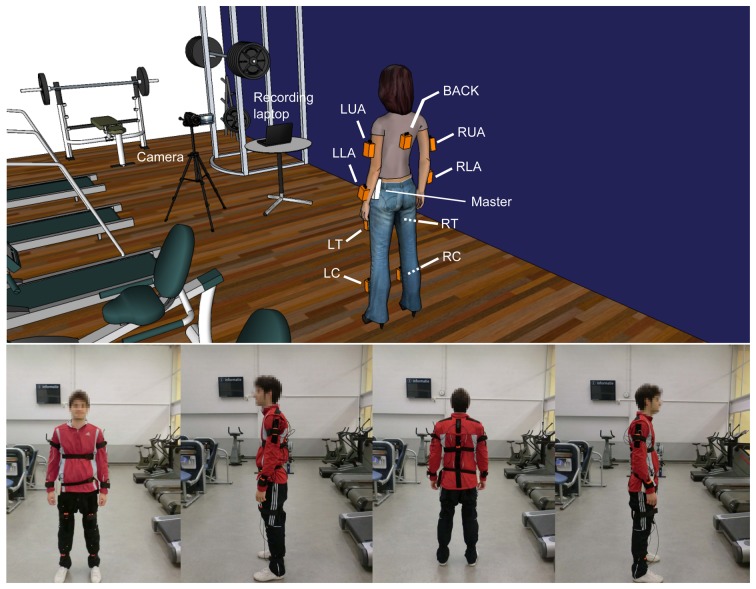
Experimental setup (cardio-fitness room). Eight Xsens units are placed on each body limb and an additional one on the back. A laptop is used to store the recorded data and for labeling tasks. A camera records each session for offline post-processing. Sensor legend: left calf (LC), left thigh (LT), right calf (RC), right thigh (RT), back (BACK), left lower arm (LLA), left upper arm (LUA), right lower arm (RLA) and right upper arm (RUA).

The study setup is depicted in Figure 3. A set of nine inertial measurement units (Xsens MTx, [30]) are distributed on the subject's body. These nodes provide several sensing modalities, including acceleration, rate of turn and magnetic field, and can also derive the spatial orientation estimates of the sensor frame with respect to the Earth reference. Individual sensors and the Xsens Master processing unit are wired together in a serial connection that nevertheless does not limit users' mobility. The Xsens Master device is interfaced over Bluetooth to a laptop, which continuously records the information delivered by the nodes. The laptop is also used by the experimenter for online labeling of the activities. Both data storage and labeling processes are performed using the Context Recognition Network Toolbox (CRNT) [31]. The sampling rate is set to 50 Hz, which is considered sufficient for capturing human activity.

Eight of the sensors are normally positioned on the middle of each limb (for each extremity). An additional one is centered on the back, slightly below the scapulae. The sensors were attached to the body using elastic straps and velcro. Trousers and sports jackets of different sizes were provided in order to ensure the fit to the user.

All sessions were recorded using a video camera. The video recording is useful to check anomalous or unexpected patterns in the data and to correct labeling mistakes. In some recordings, two subjects performed the exercises in parallel for efficiency.

The experiments took place in a cardio-fitness room at the Student Sport Centre, Eindhoven. The recordings were performed for 17 volunteers, seven females and ten males, with ages ranging from 22 to 37 years old. The experiment consisted in performing the complete set of exercises twice, once with the self-placed and once with the default sensor setup. The self-placed run was performed first, so as to not give any clues about the default sensor position to the participant. One run-through of the exercises lasted 15–20 min. Each session was preceded by a preparation phase lasting around 30 min. For the self-placement recordings, first, the users self-positioned three out of the nine sensors, while the remaining sensors were attached by the specialist on their predefined placements. This is considered a reasonable estimate of the proportion of sensors that may be misplaced during the normal wearing of the devices. For the second run, self-placed sensors are relocated in their default placements. The preparation phase also comprised the connection of the sensors to the Xsens Master, setting up the video camera and establishing the Bluetooth connection between the Xsens Master and the laptop. Before starting the exercises, the exact positions of the sensors were documented using the video recording.

Three out of the 17 volunteers were recorded for the induced-displacement scenario (concretely, Subjects 2, 5 and 15). In contrast to the self-placement case in which the inter-subject variability is intended to be observed when self-placing the sensors, this scenario rather focuses on the study of the effects of large displacements that are intentionally introduced. For this setup, the instructor misplaced a subset of the sensors, while maintaining the rest in their original location. Data for various sensor configurations were recorded, concretely for the case in which four, five, six or even seven out of the nine sensors are misplaced. The participants completed a run for each sensor configuration. For both self-placement and induced-displacement scenarios, the displaced sensors were selected to approximately cover all body parts.

An instructor demonstrated each exercise before the user performed them, although the participants were asked to freely execute the activities while trying their best. In general, 20 repetitions were recorded for each activity, except for those exercises that required gym machines (*i.e.*, L1–L3 and L31–L33 from Table 1), for which roughly a minute of exercising was recorded. This provided a way to obtain a sufficient number of activity instances for each class. The two runs were separated by a break, during which the battery levels were checked and the setup for the next run prepared. The exercises were labeled online using the CRNT. The inevitable errors in the online labeling were eliminated in post processing with the help of a commentary sheet to track the errors while recording, in addition to the video recording.

Table 2 presents the statistics on the available exercise data with respect to the complete recorded data for each displacement concept considered in this work. The entire dataset contains over ten hours of exercise data and lasts over 39 h in total. Self-placed and ideal recording sessions contain approximately 15 h of data each. Induced-displacement sessions include more than ten hours of data distributed among the different runs. The difference between exercise duration and the total duration provides the amount of data corresponding to unrelated or non-target activities. The average ± standard deviation duration in minutes of the exercise data (total data) recorded per subject is 13.02 ± 5.26 (51.31 ± 20.35) for the ideal concept, 13.96 ± 3.78 (50.59 ± 17.41) for the self-placement scenario and 14.75 ± 5.36 (49.24 ± 22.43) for the induced case.

**Figure 4.**
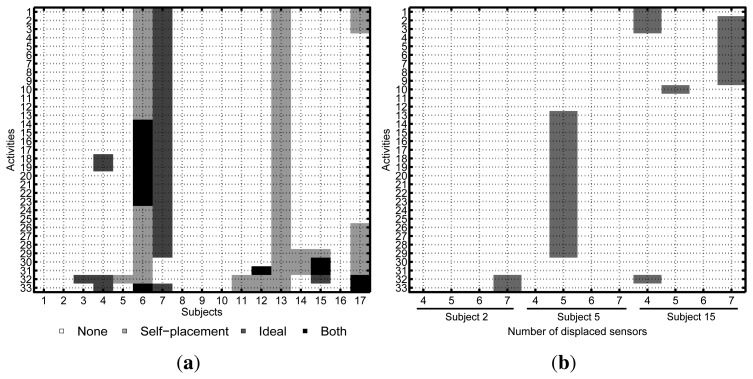
Missing activity data for each particular subject (shading spots). (**a**) For ideal and self-placement conditions: the legend identifies the corresponding sensor deployment (both ≡ self-placed and ideally-placed). (**b**) For the induced-displaced condition: only Participants 2, 5 and 15 were considered.

**Figure 5.**
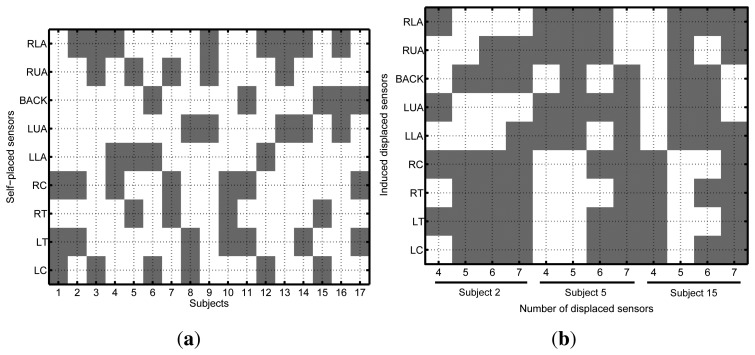
Shading spots identify the displaced sensors for the (**a**) self-placement and (**b**) induced-displacement deployments. Only Participants 2, 5 and 15 were considered in (**b**).

During the data post-analysis, some parts of the recordings were identified as either corrupted or missing. The recorded videos proved to be especially useful for rejecting erroneous labels, as well as checking the validity of the annotated data. Figure 4 shows the missing activity data for each subject or run. No activity data is available for Subjects 6 and 13 for the self-placement setup. For Participant 7, there is almost no data available in the ideal scenario. A few additional activities are missing for some of the remaining subjects. For the induced-displacement dataset (Figure 4b), the worst data loss was incurred for Subject 3, where activities L13 to L29 are missing. A few additional activities are also missing for the rest of the subjects. In either case, the amount of missing data is negligible compared to the number of valid samples; thus, it was decided not to redo the corresponding experiments. Finally, the sensors that have been displaced for each subject and setup are depicted in Figure 5.

## 5. Methods and Results

This section analyzes the tolerance of activity recognition systems to the effects of sensor displacement measured in realistic settings. Common single and multiple sensor configurations are evaluated for this purpose. Firstly, the activity recognition methods considered in this work are described in the following section.

### 5.1. Activity Recognition Methods

The activity inference process, also referred to as the activity recognition chain (ARC) [32]), consists of a set of specific steps that mainly combine signal processing, pattern recognition and machine learning techniques to implement a specific activity recognition system. Concretely, a set of sensors usually deliver raw unprocessed signals, which represent the magnitude of the measured physical quantity (e.g., acceleration). The registered information may be disturbed by electronic noise or other kinds of artifacts. Depending on whether a certain information loss is tolerated, sometimes the signals are preprocessed through filtering techniques [33,34]. In order to process the continuous signal streams, these are partitioned into segments of a fixed or variable size. Different techniques are utilized for that purpose, mainly based on windowing, event-based or activity-defined segmentation [35]. Subsequently a feature extraction process is carried out on each data window to provide a handler representation of the signals for the pattern recognition stage. A wide range of heuristics [36], time-frequency domain [37,38] and other mathematical and statistic functions [39] are commonly used. In some cases, a feature selector is used to reduce redundancy among features, as well as to minimize dimensionality. Examples of feature selection methods used in activity recognition are principal or independent component analysis [40], forward-backward selection [41] or correlation [37]. The resulting feature vector is provided as the input of a classifier, which ultimately yields the recognized activity or class to one of those considered for the particular problem. Extensive topical reviews of the activity recognition classic methodology can be seen in [42,43].

Diverse variants of the general ARC are normally implemented depending on the employed sensor topology. Activity recognition systems may build on data measured through a sole sensor, thus implementing a single ARC (SARC). These are the systems most widely employed by current vendors of activity recognition applications, for example as part of smart clocks [44,45], bracelets [46], fitness bands [47,48] or clips [49]. Capturing the motion of different body parts normally enhances the system recognition capabilities. This can be performed through multi-sensor configurations, which are less habitual in commercial devices. For that case, activity recognition systems need to fuse the information coming from each sensor node. This may be performed at the feature level, *i.e.*, by aggregating the features extracted from each sensor data stream before input to a single classifier [50] (feature fusion multi-sensor ARC (FFMARC)), or at the classification level, *i.e.*, by combining the decisions of independent classifiers built on the data of each respective sensor (decision fusion multi-sensor ARC (DFMARC)). For the DFMARC case, a novel approach presented in a recent work [51], namely the hierarchical weighted classifier (HWC), is used. This model is considered here, since it was already proven to provide a relevant tolerance to synthetically modeled sensor displacement [22].

### 5.2. Experimental Setup

Diverse scenarios are defined to analyze the impact of the activity recognition problem complexity on the robustness of the systems to sensor displacement. Two reduced versions of the original dataset, *i.e.*, comprising a subset of the whole group of activities, and the actual original dataset are used for evaluation. Specifically, these datasets respectively embrace 10 activities, 20 activities and 33 activities (all) from the original set. To ensure a fair distribution of the diverse type of activities registered for this dataset, exercises that involve the motion of different parts of the users' body are selected for the 10-activities and 20-activities datasets. The selected exercises are *1*, *4*, *8*, *10*, *12*, *18*, *22*, *25*, *28* and *33* for the 10-activities scenario and *1*, *2*, *3*, *7*, *12*, *13*, *17*, *18*, *19*, *20*, *21*, *23*, *25*, *27*, *28*, *29*, *30*, *31*, *32* and *33* for the 20-activities case (see Table 1 for the equivalence). The data corresponding to these activities are chosen for the diverse concepts of sensor placement and displacement (“ideal”, “self” and “induced”).

For the SARC models, the activity recognition processing is performed on the data registered through each sensor in an independent manner. The FFMARC and DFMARC (HWC) models make use of the data registered through the complete ecology of sensors. Acceleration data are particularly utilized, since this sensor modality is predominantly used in activity recognition applications. According to the ARC, a segmentation process consisting of a non-overlapping sliding window (6-s size, as suggested in [23]) is applied to each data stream. Three feature sets (FS) are respectively extracted for evaluation: FS1 = “mean”, FS2 = “mean and standard deviation” and FS3 = “mean, standard deviation, maximum, minimum and mean crossing rate”. These are features widely used in activity recognition [23,38,52–54] for their discrimination potential and ease of interpretation in the acceleration domain. Additionally, the use of these features may also simplify the task of reproducing these experiments for future work comparison. Likewise, three of the most extensively and successfully used machine learning techniques in previous activity recognition problems are considered for classification: C4.5 decision trees (DT, [55]), k-nearest neighbors (KNN, [56]) and naive Bayes (NB, [57]). The *k*-value for the KNN model is particularly set to three, as it has been proven to provide good results in related works and for different settings [22,51,58]. These techniques are used for the standard classifiers (SARC and FFMARC) and for the HWC base classifiers.

The evaluation of the activity recognition models for the “ideal” settings is performed through a ten-fold random-partitioning cross-validation process applied across all subjects and activities. This provides the baseline recognition capabilities of these systems in absence of displacement. This process is repeated 100 times for each method to ensure statistical robustness [59]. A testing procedure is applied for “self” and “induced” settings. Thus, self-placement and induced-displacement data is inputted to a predefined activity recognition system, which is obtained from the training on the data registered for the ideal-placement setting.

### 5.3. Single Sensor Performance

**Figure 6.**
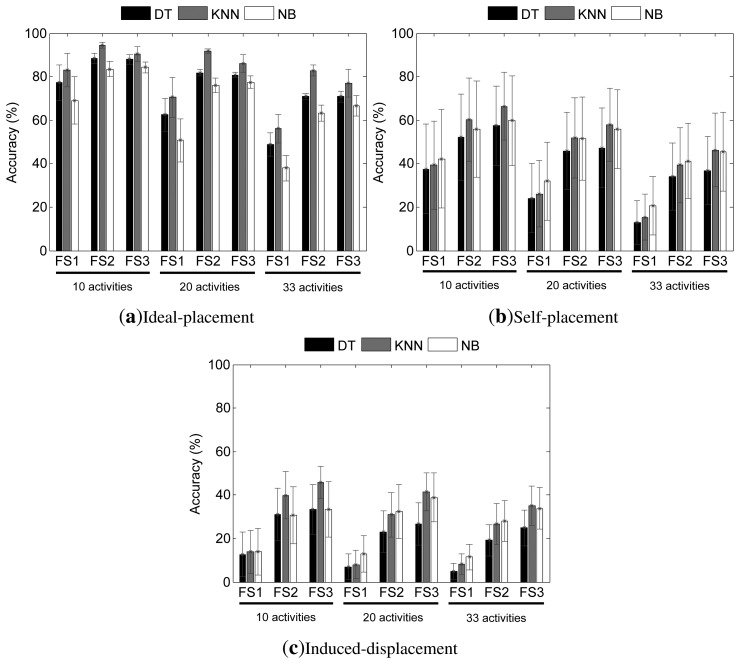
The accuracy (average bar and standard deviation whiskers) results from the evaluation of the single sensor approach across all subjects and sensors for the (**a**) ideal-placement; (**b**) self-placement and (**c**) induced-displacement settings. The top legend identifies the classification paradigm. The horizontal axis labels identify the feature set used for each experiment. The activity recognition dataset (*i.e.*, the number of activities) used is respectively underlined.

In this section, the results corresponding to the evaluation of the SARC systems are presented. The performance results obtained from the evaluation across each individual sensor are depicted for each displacement-concept setting in Figure 6. Particularly, the accuracy rate is used as a performance measure, which defines as the proportion of correct classifications with respect to the total classified instances.

In general terms, two tendencies could be discerned for all settings and scenarios. First, as the complexity of the problem increases, *i.e.*, as the number of considered activities grows, the detection performance reduces. Second, the use of richer feature sets helps improve the recognition capabilities of the systems; thus, the best results are normally obtained for FS3, followed by FS2 and FS1.

For the ideal-placement (Figure 6a), from all evaluated models, the KNN stands out, with values above 90% accuracy for the 10-activities and 20-activities problems. The performance drops to a bit more than 80% for the 33-activities scenario. The other two paradigms show limited applicability for recognition, especially for the most complex scenarios.

A significant drop on the recognition capability is seen when the users self-place the sensors (Figure 6b). Through comparing these results with the ones obtained for the ideal case, for the simplest scenario (*i.e.*, 10 activities) a drop of approximately 40% accuracy is observed for the KNN approach and FS1, that nevertheless gets reduced to almost 35% and 25% drop for FS2 and FS3, respectively. This “enhancement” for FS2 and FS3 could be explained, since the use of richer feature vectors may help compensate the sensitivity to displacement variations of the features used in FS1. The performance gap is higher for the 20-activities and 30-activities scenarios, in which the accuracy decreases by up to 45%. The tendencies observed for the KNN are similarly applicable to the DT model. On the other hand, NB appears to be the most robust approach. The drops are very similar for all scenarios (around 20%–25%, the lowest evaluated). In fact, the performance for NB after displacement is quite similar to that of the KNN model. In either case, since the maximum performance across all models and paradigms is of 65%, it can be concluded that none of the models satisfactorily cope with the displacement introduced by the users when self-placing the sensors. In other words, these models are not practical for recognition purposes under these circumstances.

Finally, for the induced-displacement setting, the data corresponding to the displaced sensors of all categories (four, five, six and seven displaced sensors) are used for evaluation. Similarly, as in the self-placement setting, an overall analysis of the effects for each sensor (*i.e.*, body location) is feasible here, since most possible combinations of displaced sensors were considered during the dataset recording process (see Figure 5). From Figure 6c, the tremendous drop in performance could be observed that the diverse recognition systems suffer under the assumption of an intentional mispositioning of the sensors. Clearly, these are the poorest results among the three settings, with performance values that are below 50% accuracy at best. As for the self-placement setting, the minimum performance decrease is encountered for the NB model, while the highest drop is found for the KNN technique, although this yields the best performance values from the evaluated models.

### 5.4. Multi-Sensor Fusion Performance

**Figure 7.**
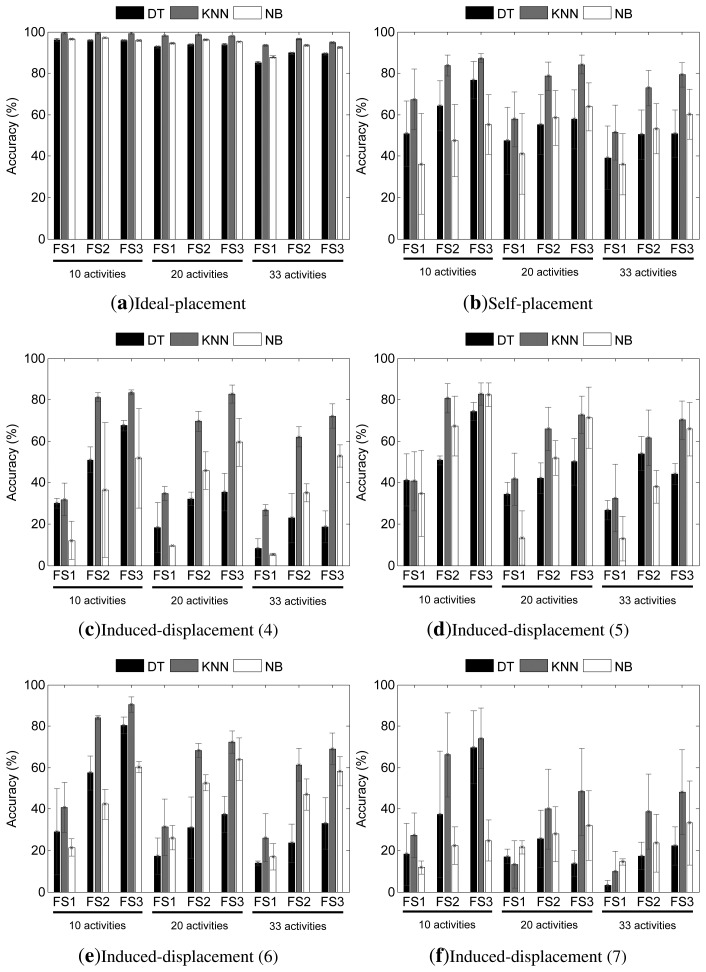
Accuracy (mean and standard deviation) results from the evaluation of the feature fusion model for the (**a**) ideal-placement, (**b**) self-placement and (**c–f**) induced-displacement (number of sensors) settings. The top legend identifies the classification paradigm. The horizontal axis labels identify the feature set used for each experiment. The activity recognition dataset (*i.e.*, the number of activities) used is respectively underlined.

**Figure 8.**
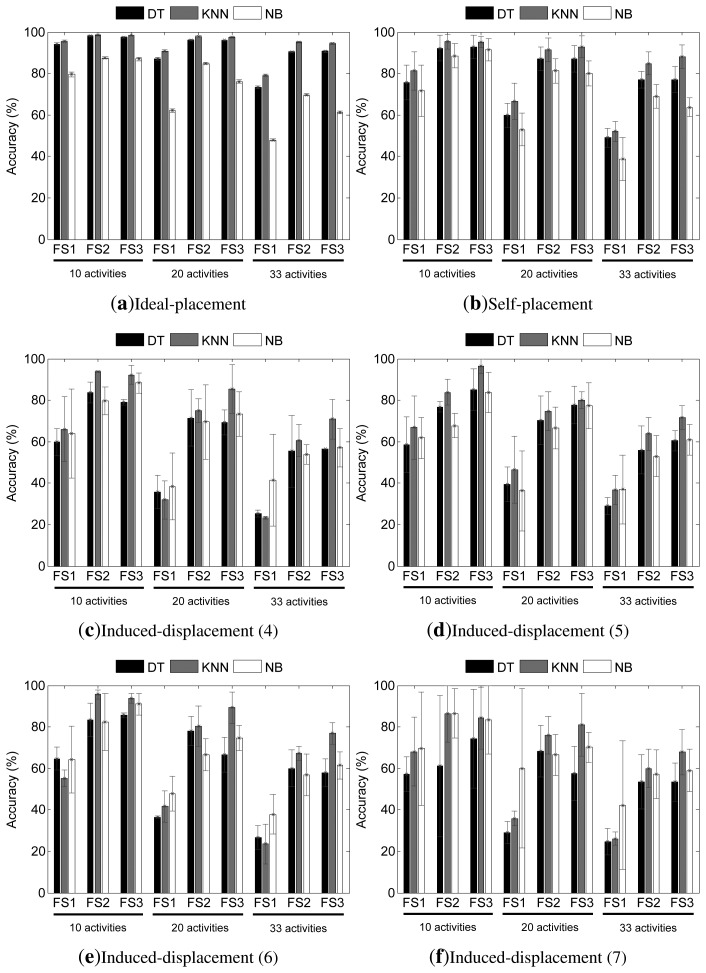
Accuracy (mean and standard deviation) results from the evaluation of the decision fusion model for the (**a**) ideal-placement; (**b**) self-placement and (**c–f**) induced-displacement (number of sensors) settings. The top legend identifies the base classifiers paradigm. The horizontal axis labels identify the feature set used for each experiment. The activity recognition dataset (*i.e.*, the number of activities) used is respectively underlined.

The accuracy results for both feature fusion (FFMARC) and decision fusion (HWC) approaches are respectively depicted in Figures 7 and 8. It should be noted that unlike during the individual sensor analysis, here, all of the sensors are simultaneously used, then the evaluation could be respectively performed on the diverse categories devised for the induced-displacement setting (*i.e.*, four, five, six or seven out of the nine sensors are mispositioned).

Similarly to what was commented on for the individual sensor approach, it could be seen that the performance of the diverse recognition systems improves as the number of features grows. On the other hand, the performance decreases as the activity detection problem gets more complex (*i.e.*, the number of activities increases), albeit this performance drop is less marked than for the SARC models.

The feature fusion model shows high recognition capabilities for all activity recognition scenarios when an ideal placement of the sensors is considered (Figure 7a). In fact, the recognition performance is near perfect for models implementing the KNN technique, with an accuracy of more than 95% for the most complex scenario. Furthermore, very high figures are obtained for the other two classification methodologies. DT proves to be the least reliable of all of the methods, yet it provides performance values above 90% for the 10- and 20-activities scenarios. Finally, NB yields accuracies that span from 87% for the most complex scenario and FS1, to 97% for the simplest scenario and FS2 or FS3.

Different readings can be extracted for the self-placement case (Figure 7b). Here, the KNN model stands out as the most reliable technique and also the most robust, as this suffers from the lowest performance decrease with respect to the default setup. Despite the fact that there is a moderate drop for the simplest activity recognition scenarios when rich feature sets are used (84% and 87% accuracy for the 10- and 20-activities while using FS3), a considerable drop is found for the most complex scenario, which leads to average top performances of less than 80%. The highest drops could be seen for the use of FS1 as the feature input, with average performance values that reach 70% accuracy at best and for the simplest scenario, to almost 50% for the 33 activities case. In summary, the performance decrease approximately spans from 13% at best to more than 40% at worst. The worsening is even higher for DT and NB, with performance drops that range between 20% and 60%.

The effects of the induced-displacement are shown to be in general more harmful than for the self-placement case. Similarly, as for the ideal and self-placement cases, KNN proves to be the most accurate model in general terms. Nevertheless, the performance decreases differently for each scenario. Starting with the case in which four sensors are intentionally mispositioned (Figure 7c), it can be observed that a maximum accuracy above 80% is guaranteed for FS3 and 10- or 20-activities. This represents a reduction of approximately 20% with respect to the ideal case. For the rest of the evaluations, the drop is very significant, decreasing in some cases up to 90% with respect to ideal circumstances. When five sensors are displaced, the performance drops span from 20% to 85% at worst.

An improvement is observed for the case of mispositioning six sensors for the 10-activities scenario, although the performance worsens for the other two scenarios (up to a 75% loss). Finally, as could be expected, the worst results are obtained when seven out of the nine sensors are displaced. In average terms, none of the models gets a performance superior to 75%, especially for the most complex scenarios, in which the highest drop is about 85%. To explain the singularity found for the six-sensors-displaced setting (better overall results for the simplest scenario than for four and five), it must be borne in mind that for the induced-displacement evaluation, the recordings for just three participants are available; thus, the conclusions could be, in principle, less generalizable than for the self-placement case. Moreover, some data are missing for part of the users, as illustrated in Figure 4b, particularly for one of the volunteers that participated in the induced-displacement (5) recordings, which may also explain these trends. The differences among these could be also explained through the random nature of the displacement introduced from run to run; thus, larger sensor displacements may be present for this setting than for others. In either case, this idea fits well with what can be observed in realistic settings.

According to the HWC model, very promising results are obtained in ideal settings for all scenarios, although this especially applies to DT and KNN models. Accuracy rates above 90% and 95% may be respectively obtained for the DT and KNN models for all scenarios and features sets, albeit these reduce for the most complex scenarios when the simplest feature set is used (FS1). The NB technique proves to be the least reliable, with performance values that improve when richer feature sets are used. Although performances over 87% and 85% could be obtained for the 10- and 20-activities scenarios, only a 70% accuracy may be achieved for the 33-activities case when using the NB technique.

The HWC model significantly overcomes the worsening experienced by the SARC and FFMARC models when the user self-places the sensors, especially for the most complex scenarios. Similarly, as for the feature fusion model, KNN is once again the most promising technique to be used as a base classifier. For this, when FS2 and FS3 are used, the performance achieved is superior at 95% for the 10-activities scenario, 92% for the 20-activities case and close to 90% for the 33-activities scenario and FS3. This translates into very reduced performance drops that span from 3% to 6% at worst. As it happens to occur to the SARC and FFMARC models, the approaches that utilize the FS1 are not capable of dealing with the effects of displacement; however, the drops are yet lesser than for the single sensor and feature fusion models. The DT and NB models are shown to be quite robust considering their moderate performance drops, which, in most cases, are as reduced as for the KNN approach. This clearly supports that such robustness is due to the HWC structure, since the behavior is quite similar with the independence of the machine learning paradigm used for the base classifiers.

The combination of HWC and KNN turns out to be the most reliable model under the assumption of induced displacements (Figure 8c–f). NB, on the other hand, is the approach that, while not yielding the best average performance, suffers from the lowest performance drop. From an individual observation, performance rates of up to 94% may be achieved for the simplest scenario when four sensors are mispositioned. This performance decays to 85% and 70% for the 20-activities and 33-activities scenarios, quite in line with the results obtained for the feature fusion, which here nevertheless correspond to a lower performance drop. Concretely, the performance drops for this case range from 5% to 35% for KNN and FS2 or FS3 and reach a 60% drop when FS1 is used. For NB and DT, the decrease is found to be even lower, with a performance improvement only shown for a few cases. The evaluation on the data pertaining to the mispositioning of five sensors gives results that show no deterioration of the performance for KNN-FS3 and 10-activities, while a significant worsening is seen for the most complex scenarios. Particularly, the performance decrease ranges between 2% and 45% for this setting. Slightly better results are obtained for the six-sensors-displaced case, in which accuracies of more than 90% are reached for the 10- and 20-activities scenarios, while little more than 75% could be achieved for the 33-activities problem. Overall, the performance drop spans from 3% at best to 55% at worst. Finally, the worst results are obtained for the seven mispositioned sensors setting. Again, NB proves to be the most robust technique, whereas the maximum performance is generally obtained by KNN. The performance drop ranges from 2% to more than 55%. The high standard deviation values for this last setup reflect the differences about the deployment used for each user and run.

## 6. Discussion

In general terms, two tendencies could be discerned for all results independently of the sensor deployment settings. First, as the complexity of the problem increases, *i.e.*, the number of considered activities grows, the detection performance reduces. Second, as was expected, the use of richer feature sets helps to improve the recognition capabilities of the considered systems; thus, the best results are normally obtained for FS3, followed by FS2 and FS1.

The accuracy results prove that the evaluated activity recognition approaches could be adequate solutions under the assumption of an invariant sensor deployment. Nevertheless, both sensors' self-placement and induced-displacement settings introduce a significant drop on the performance of standard activity recognition systems with respect to an ideal sensor deployment, which confirms the effects described in Section 3. Clearly, the more profound the displacement applied, the higher the loss in performance. Likewise, the more sensors are mispositioned in a multi-sensor setup, the more limited the recognition accuracy is. The HWC model proves to be the most robust approach from all evaluated systems, capable of dealing with the variations introduced during the user self-placement of the sensors and also overcoming the impact of large mispositioning for particular scenarios.

### 6.1. Single vs. Multiple Sensing

For the single sensor-based approach, KNN appears to be the most reliable technique in ideal circumstances, but possibly the most sensitive to sensor displacement. This could be a consequence of the way the activity clusters drift in the feature space because of the displacement effects. The use of larger *k*-values may help to increase the tolerance of this algorithm to drifts in the feature distributions. On the contrary, models based on NB appear to be the less accurate for predefined conditions, although these suffer from the lowest drop on the performance when the sensors are misplaced. In any case, the maximum performance obtainable in the event of sensor displacement does not suffice for the purpose of activity recognition, thus, once again, demonstrating the potential limitations of the SARC approaches in realistic settings.

Models based on a multi-sensor setup generally allow for accurate recognition capabilities given a default deployment of the sensors. Besides, more promising results are obtained under the effects of sensor displacement than for the individual sensor approach. Nevertheless, not all models exhibit the same tolerance to sensor misplacement. In fact, the FFMARC model shows an important worsening for the case in which the user self-places the sensors. The performance drop is even higher when the sensors are purposely misplaced. For the FFMARC model, NB proves to be the most sensitive to sensor drifts, while the opposite is observed for the k-nearest neighbor technique.

On the contrary, the HWC model deals quite well with the effects of sensor displacement introduced by a user self-placement of the sensors. The HWC model also exhibits a high tolerance to large misplacement for simple activity recognition scenarios. This is shown to happen independently of the classification paradigm utilized for the base classifiers. However, as the number of displaced sensors increases, the performance of both FFMARC and HWC approaches generally declines. The recognition capabilities are especially reduced when a majority of the sensors are displaced and for very complex recognition problems. Even though, comparatively, here, again, systems based on the HWC scheme significantly overcome the robustness capacity of FFMARC models.

### 6.2. Hierarchical Weighted Classifier (HWC) Advantages

HWC assures a higher robustness to sensor displacement effects than standard activity recognition approaches. These better results for the HWC may be explained, since individual variations of a sensor with respect to its default behavior have less impact on the classification process. This is possible, since each sensor contributes in an independent manner to the final delivered decision; thus, a majority of sensors (normally unaffected) overcome the decisions obtained from the displaced sensors. Conversely, single sensor models and feature fusion models incorporate data from displaced sensors in a unique feature vector, thus leading to a potential feature drift that cannot be handled by the reasoning model.

The HWC model shows an important tolerance to sensor displacement, although it is subject to a certain performance drop. However, it is worth noting that this drop applies homogeneously to each classification paradigm, which leads us to conclude that the potential robustness of the system truly relies on the HWC structure more than the specific base classifiers. In this way, the HWC model proves to increase the classification potential and robustness of standard multi-class models.

Given the novelty of this approach, it is not possible to compare the results provided with prior work. As shown in Section 2 and to the best of our knowledge, there is no investigation that treated on-body sensor displacement in this regard, *i.e.*, analyzing the effects of realistic sensor displacement generated by users' self-placement and large misplacement.

### 6.3. Concepts of Displacement

This is the first study in this field that widely accounts for the problem of sensor self-placement in the investigation of real-world activity recognition issues. As proven through this work, there are serious consequences when allowing users to place the sensors by themselves. Activity recognition systems based on a single sensor may be useless if the user does not follow the specific deployment instructions. This is a limitation that goes against usability requirements of real-world activity recognition systems. Sensors are devised to be embedded in the articles of daily living; thus, restricting the way these must be worn can prove burdensome and could reduce users' acceptance. When wearing multiple sensors, the effect of isolated sensor misplacements may be reduced. In fact, it could be envisioned that a bracelet is worn on the upper arm instead of the lower arm, but it is normally expected that a majority of other instrumented items, such as shoes, trousers or shirts, are put on in a predefined manner. In this direction, the HWC model helps overcome the limitations introduced by a minority of drifted sensors.

The induced-displacement setting was originally planned to analyze the effects of a large misplacement of the sensors. This could be also part of the day-to-day usage of the systems, but it is rather expected to be seen in occasional circumstances. In real-world settings, this could apply to sensor displacements, such as those introduced when rolling up the sleeves of an instrumented shirt or when wearing the shirt back-to-front. In wearable activity recognition, this type of displacement has been hardly studied, but for a particular body part and in a very limited way in terms of the number of users and activities. In this work, several configurations and settings are analyzed, thus bringing added value with respect to the state-of-the-art. This study also proves the worsening of the recognition capabilities as the number of displaced sensors increases. Neither individual sensor approaches nor feature fusion models are found to be capable of overcoming the challenge of multiple displaced sensors. The HWC model partially deals with this, but for the simplest scenarios.

### 6.4. AR Problem Complexity

In general, as the complexity of the recognition problem grows, an accurate recognition of the diverse activities turns out to be more difficult. Something similar is observed under the assumption of sensor displacement. Concretely, for the ten activities scenario, low performance reductions are seen, especially when the HWC and richest feature sets are utilized. However, as the number of activities grows, also the effects of displacement are more prominent, and by extension, also, the recognition worsening is more profound. This may be explained, since the probability of misclassification of a given instance is higher, because there are more potential activity clusters with which this may be confused. The use of more features or kernel-type transformations of the feature space could be of interest to separate as much as possible the diverse class clusters and, therefore, reduce the activity confusion likelihood.

### 6.5. Study Generalization

One of the key elements that supports the generalization of the conclusions of this study is the data employed for evaluation. The considered sensor deployments comprise some of the most widely used placements in wearable sensing. Sensors are located in unobtrusive and comfortable positions that permit the normal behavior of users during their daily living. Moreover, the deployment almost covers the complete body, concretely registering the movements of all limbs and trunk. To be perfectly complete, sensors on the head should be mounted; however, this option was dismissed, considering its rare application, normally motivated to avoid users' discomfort. In terms of activities, a broad number of exercises of diverse type are considered. This includes exercises of different intensity and velocity that also involve diverse combinations of body parts. This helps to gain insights into the effects of sensor displacement for activities in which some body parts are still or quasi-static, to others in which these are in motion, thus tackling a wide spectrum of activities. A considerable number of participants were also considered for the recordings, thus better supporting the validation of the study outcomes. Although participants of a relatively similar age were considered for evaluation, their physical conditions were quite diverse, thus ensuring different kinds of executions. This is further supported through letting the users execute the activities in a natural way.

For the sake of generalization, the recognition systems tested here correspond to some of the most widely used in related works. Moreover, simplicity and comprehensiveness were key elements considered during the selection of these models, thereby allowing us to focus on the potential impact of the displacement effects. Thus, for example, data directly captured through the sensors are used, avoiding any kind of filtering or preprocessing. These procedures normally remove some parts of the raw signals that may potentially lead to a change in the signal space. This change could mask the actual effects of sensor displacement. Moreover, the features used are simple, easy to calculate and with interpretable physical meaning. Concretely, the “mean” allows us to extract the gravitational component contribution to the acceleration, which is particularly informative for distinguishing among low intensity activities. “Standard deviation”, “minimum” and “maximum” provide insights into the intensity and magnitude of the movements, while the ‘mean crossing rate’ correlates with the dynamicity and frequency of the executions. Similar tendencies have been found for the various feature sets for each independent classification methodology, thus demonstrating that the results obtained here could be extrapolated to other systems of a similar nature. In either case, the differences among performance quality for each feature set determine that an automatic selection of better features could possibly lead to improved results.

### 6.6. Open Issues

The aim of this work was to analyze the effect of static displacements normally introduced during sensors placement or relocations. These displacements remain in principle static during the use of the systems, although small variations of sensor positioning are sometimes observed, because of loose attachments or when performing the activities. This allows us to further extend some of the conclusions to the case of dynamic displacements. Nevertheless, a more profound investigation in this direction is required to better determine the specific effects of dynamic sensor displacements.

Future work may also include the evaluation of the effects of extreme sensor misplacement. Extreme sensor misplacement here refers to the displacement introduced when two or more sensors are exchanged, thus relocated in body parts completely unrelated to the position devised at the time of design. For example, it could be expected that a user misplaces a sensor during the self-placement process, consequence of a mistake (e.g., the wrist-sensor is positioned on the ankle). Although less frequent, real-world activity recognition systems should account for these extreme sensor misplacements, thus contributing to a less constrained use of the devices.

Similarly, for the self-placement setting, it would be also interesting to provide total freedom to users to place the sensors wherever they prefer. Although no hints were given to the participants on how they had to put the sensors on, indications about the particular body part were provided. It was necessary to complete the sensor deployment with the default placement of the remaining sensors. Therefore, a study in which the users self-attach the complete set of sensors in an arbitrary manner could be of utility.

When a majority of the sensors are mispositioned, and especially for complex scenarios, the proposed models are not capable of dealing with the effects of the displacement. Considering the structure of the HWC model, an interesting approach would be to automatically update the parameters of the model to reduce the impact of the decisions yielded by the displaced sensors. To that end, ascertaining which sensors have been displaced is completely necessary. This could be performed through the use of collectivity to identify which sensors are anomalously behaving or through statistical analysis of the variations of each sensor data stream.

## 7. Conclusions

Restricting the way in which users must place their wearable sensor devices is unrealistic, unpractical and contributes to people's lack of interest in the use of these systems. In fact, when these sensors are considered to be potentially embedded in clothes, garments or other portable accessories of daily living, the casualness and naturality with which users normally put on these items must seriously be taken into account. When a sensor is placed in a different position with respect to its ideal or default placement, an effective displacement could be identified from the former to the latter position. This sensor displacement, which could be categorized as a combination of rotations and translations, normally translates into a drift of the original signal distribution, a variation that further propagates along the complete activity recognition process. This potentially leads to a malfunctioning of activity recognition systems designed to operate on a definite sensor deployment.

This work focused on the effects of displacements introduced during normal use of the sensors, particularly when wearing the devices. To that end, a novel open-access benchmark dataset is used for the study of this problem. A concept for categorizing inertial sensor displacement conditions in ideal-placement, self-placement and induced-displacement is introduced. Ideal-placement settings correspond to the case in which the sensors are positioned according to the default sensor deployment devised during the design phase, thus representing a recognition baseline for default conditions. Self-placement settings reflect a user-centered observation of how people place the sensors by themselves, e.g., in a sports or lifestyle application. Induced-displacement conditions correspond to extreme displacement variants and, thus, could represent boundary conditions for recognition algorithms.

The results obtained in this work show that the way users place the sensors in a natural manner could have serious consequences on the system's recognition capabilities. A considerable loss of performance is observed in most evaluated activity recognition systems, especially those based on a single sensor unit. The use of multi-sensor configurations helps to counteract the variations introduced in the sensor deployment. Nevertheless, not all models demonstrate the same tolerance to sensor displacement. In fact, the performance of feature fusion approaches is subject to a severe worsening for the case in which the user self-places the sensors. The performance drop is even higher when the sensors are intentionally misplaced. Feature fusion multi-sensor models severely suffer from the effects of displacement, since changes in the signal space are naturally incorporated into the aggregated feature vector. Neither single sensor nor feature fusion multi-sensor approaches demonstrate acceptable recognition capabilities after displacement. On the contrary, the proposed hierarchical weighted classifier (HWC) shows a notable tolerance to sensor displacement, standing out as the most robust approach from the evaluation. This is possible because, in this model, the decisions are considered independently and the collectivity copes with the errors introduced by displaced sensors. The performance drop is more significant when a majority of sensors are displaced in an extreme fashion. In this case, only the HWC model shows accurate recognition, but for the simplest activity recognition scenario.

## Figures and Tables

**Table 1.** Warm up, cool down and fitness exercises considered for the activity set. In brackets are the number of repetitions (N×) or the duration of the exercises (min).

**Activity Set**	
L1: Walking (1 min)	L18: Upper trunk and lower body opposite twist (20×)
L2: Jogging (1 min)	L19: Lateral elevation of arms (20×)
L3: Running (1 min)	L20: Frontal elevation of arms (20×)
L4: Jump up (20×)	L21: Frontal hand claps (20×)
L5: Jump front & back (20×)	L22: Frontal crossing of arms (20×)
L6: Jump sideways (20×)	L23: Shoulders high-amplitude rotation (20×)
L7: Jump leg/arms open/closed (20×)	L24: Shoulders low-amplitude rotation (20×)
L8: Jump rope (20×)	L25: Arms inner rotation (20×)
L9: Trunk twist (arms outstretched) (20×)	L26: Knees (alternating) to the breast (20×)
L10: Trunk twist (elbows bent) (20×)	L27: Heels (alternating) to the backside (20×)
L11: Waist bends forward (20×)	L28: Knees bending (crouching) (20×)
L12: Waist rotation (20×)	L29: Knees (alternating) bending forward (20×)
L13: Waist bends (reach foot with opposite hand) (20×)	L30: Rotation on the knees (20×)
L14: Reach heels backwards (20×)	L31: Rowing (1 min)
L15: Lateral bend (10× to the left + 10× to the right)	L32: Elliptical bike (1 min)
L16: Lateral bend with arm up (10× to the left + 10× to the right)	L33: Cycling (1 min)
L17: Repetitive forward stretching (20×)	

**Table 2.** Dataset description summary. The overall cumulative duration for the complete set of activities with respect to all of the collected data is given in minutes.

**Deployment**	**#Subjects**	**#Anomalous Sensors**	**#Activities Duration/Total Duration**
*Ideal-placement*	17	0	226.84/860.19
*Self-placement*	17	3	220.73/895.42
*Induced-displacement*	3	{4,5,6,7}	{47.72,45.39,48.58,46.76}/632.28
